# A single nucleotide polymorphism in the Epstein-Barr virus genome is strongly associated with a high risk of nasopharyngeal carcinoma

**DOI:** 10.1186/s40880-015-0073-z

**Published:** 2015-12-16

**Authors:** Fu-Tuo Feng, Qian Cui, Wen-Sheng Liu, Yun-Miao Guo, Qi-Sheng Feng, Li-Zhen Chen, Miao Xu, Bing Luo, Da-Jiang Li, Li-Fu Hu, Jaap M. Middeldorp, Octavia Ramayanti, Qian Tao, Su-Mei Cao, Wei-Hua Jia, Jin-Xin Bei, Yi-Xin Zeng

**Affiliations:** Sun Yat-sen University Cancer Center, State Key Laboratory of Oncology in South China, Collaborative Innovation Center for Cancer Medicine, Guangzhou, 510060 Guangdong P. R. China; Department of Experimental Research, Sun Yat-sen University Cancer Center, Guangzhou, 510060 Guangdong P. R. China; Department of Medical Microbiology, Qingdao University Medical College, Qingdao, 266021 Shandong P. R. China; Department of Microbiology, Tumor and Cell Biology, Karolinska Institute, 17177 Stockholm, Sweden; Department of Pathology, VU University Medical Center, Amsterdam, 1007 MB The Netherlands; Department of Clinical Oncology, The Chinese University of Hong Kong, Hong Kong, 999077 P. R. China; Department of Epidemiology, Cancer Prevention Center, Sun Yat-sen University Cancer Center, Guangzhou, 510060 Guangdong P. R. China

**Keywords:** Epstein-Barr virus, Nasopharyngeal carcinoma, *RPMS1*, Association

## Abstract

**Background:**

Epstein-Barr virus (EBV) commonly infects the general population and has been associated with nasopharyngeal carcinoma (NPC), which has a high incidence in certain regions. This study aimed to address how EBV variations contribute to the risk of NPC.

**Methods:**

Using logistic regression analysis and based on the sequence variations at EBV-encoded *RPMS1*, a multi-stage association study was conducted to identify EBV variations associated with NPC risk. A protein degradation assay was performed to characterize the functional relevance of the *RPMS1* variations.

**Results:**

Based on EBV-encoded *RPMS1* variations, a single nucleotide polymorphism (SNP) in the EBV genome (locus 155391: G>A, named G155391A) was associated with NPC in 157 cases and 319 healthy controls from an NPC endemic region in South China [*P* < 0.001, odds ratio (OR) = 4.47, 95% confidence interval (CI) 2.71–7.37]. The results were further validated in three independent cohorts from the NPC endemic region (*P* < 0.001, OR = 5.20, 95% CI 3.18–8.50 in 168 cases vs. 241 controls, and *P* < 0.001, OR = 5.27, 95% CI 4.06–6.85 in 726 cases vs. 880 controls) and a non-endemic region (*P* < 0.001, OR = 7.52, 95% CI 3.69–15.32 in 58 cases vs. 612 controls). The combined analysis in 1109 cases and 2052 controls revealed that the SNP G155391A was strongly associated with NPC (*P*_*combined*_ < 0.001, OR = 5.27, 95% CI 4.31–6.44). Moreover, the frequency of the SNP G155391A was associated with NPC incidence but was not associated with the incidences of other EBV-related malignancies. Furthermore, the protein degradation assay showed that this SNP decreased the degradation of the oncogenic RPMS1 protein.

**Conclusions:**

Our study identified an EBV variation specifically and significantly associated with a high risk of NPC. These findings provide insights into the pathogenesis of NPC and strategies for prevention.

## Background

Nasopharyngeal carcinoma (NPC) is a malignancy with a marked geographic distribution and ethnic tendencies, occurring with high frequencies in South China, Southeast Asia, North Africa, and Alaska [1]. The etiology of NPC is complex, involving multiple factors such as genetic susceptibility, Epstein-Barr virus (EBV) infection, and environmental factors [2–4]. The known association between EBV and NPC was mainly driven by findings that EBV-encoded molecules, some of which are potentially oncogenic, were consistently observed in nearly all NPC tissues and that EBV serological markers, including viral DNA load and antibodies against viral antigens, were associated with NPC diagnosis and prognosis [5–7].

EBV infection is ubiquitous, affecting more than 95% of the worldwide population; EBV was also the first virus identified in a human tumor, i.e., Burkitt’s lymphoma. EBV has also been closely associated with Hodgkin’s lymphoma and some gastric cancers [8]. The incidences of these malignancies show remarkably different geographic distributions [9], which is paradoxical in comparison to the widespread infection with EBV. Moreover, sequence diversity in EBV genes has been demonstrated among the general population and in different tumor types [10, 11]. These results suggest the hypothesis that there might be some disease-specific EBV subtypes preferentially hazardous to certain populations, making them more prone to certain specific diseases such as NPC.

A number of studies have reported attempts to identify NPC-specific EBV subtypes using restriction fragment length polymorphism analysis and DNA sequencing based on the sequence variations of EBV genes. These genes were consistently observed in NPC tissues, including EBV nuclear antigens (*EBNAs*), latent membrane proteins (*LMP1* and *LMP2*), and EBV-encoded small nuclear RNAs (*EBERs*) [9, 10, 12]. EBV can be characterized as Type 1 (Type A) or Type 2 (Type B) based on the sequence diversity of *EBNA2* and *EBNA3s* [13, 14]. Type 1 EBV strains are more common worldwide, whereas Type 2 is equally prevalent in parts of Africa [15–17]. Based on an amino acid polymorphism at position 487 of *EBNA*-*1*, EBV has been classified into five strains: P-ala (B95-8 prototype), P-thr, V-val, V-leu, and V-pro [18–20]. V-val was detected almost exclusively in Chinese populations, whereas P-ala and P-thr were detected with a high prevalence in healthy individuals from both Chinese and non-Chinese populations [21, 22]. Based on the nucleotide sequence variations at the *LMP1* C-terminus, EBV can be separated into seven strains: China 1, China 2, Med, China 3, Alaskan, NC, and B95-8 [23]. Among the Asian isolates, China 1 and B95-8 were identified in healthy subjects, and China 1 and China 2 were found in NPC patients [23]. It has been reported that the Cantonese population is susceptible to the predominant China 1 strain in the NPC endemic region in China [24]. These investigations suggested that there were relatively stable genomic variations in EBV and that different subtypes might exist in different geographic regions.

To further identify EBV variations linked closely to NPC risk, we conducted a pilot association analysis on several important EBV-encoded genes, including *LMP1*, *EBNA1*, and the BamHI-A rightward transcripts (*BARTs*) family, starting from NPC cases and healthy controls in the Cantonese population in South China. The most striking finding is that a single nucleotide polymorphism (SNP) in the EBV-encoded *RPMS1* gene (locus 155391: G>A, named G155391A) is significantly associated with NPC incidence.

Previous studies have demonstrated that the *BARTs* family members are abnormally expressed in most NPC tissues and might contribute to NPC development [25, 26]. *RPMS1* encodes a major part of the mRNA of the *BARTs* family and is regularly transcribed in NPC tissues [26, 27]. In particular, abundant *RPMS1* mRNA was detected in NPC tissues and cell lines [28]. Considering the potential roles of *RPMS1* in NPC oncogenesis [25, 27, 29], we speculated that the sequence variation of *RPMS1* might contribute to the incidence variations of NPC among different geographic regions and ethnic groups. Therefore, we conducted a large-scale case–control study using a multistage design to identify the association between *RPMS1* variations and NPC risk.

## Methods

### Subjects and samples

For the pilot study, 60 paired NPC cases and healthy controls were recruited from Sun Yat-sen University Cancer Center (SYSUCC) between October 2005 and October 2007. Throat washing (TW) samples were subjected to polymerase chain reaction (PCR) and direct DNA sequencing to screen for genomic variations exhibiting significant differences between the cases and controls.

The discovery stage involved 346 sporadic Cantonese NPC patients and 448 healthy subjects (Data_GD1), recruited from SYSUCC and the First Affiliated Hospital of Sun Yat-sen University (1^st^ AH-SYSU), Guangdong Province, an NPC endemic region in South China, between October 2005 and October 2007.

In the validation stage, three independent sample cohorts were collected from the NPC endemic and non-endemic regions in China between October 2008 and June 2013. The first group consisted of 222 TW samples from sporadic NPC patients and 315 TW samples from healthy subjects from the SYSUCC and the 1st AH-SYSU (Data_GD2). The second group consisted of 1065 TW samples from sporadic NPC patients and 1161 TW samples from healthy subjects from the local community hospitals in Guangdong Province (Data_GD3). The third group consisted of 36 tumor biopsy (TB) samples and 66 TW samples from NPC patients from the Affiliated Hospital of Qingdao University (AH-QDU) and Shandong Province Cancer Center, in addition to 1543 TW samples from healthy subjects from the physical examination centers at local community hospitals in Shandong Province, a NPC non-endemic region in North China (Data_SD) (Table 1).

**Table 1:** Characteristics of samples from nasopharyngeal carcinoma (NPC) cases and healthy controls from the four case–control datasets

Dataset	Region	Period	NPC cases		Healthy controls		Note
			Detected/total (no.)	Source	Detected/total (no.)	Source	
Data_GD1	Guangdong	Oct 2005–Oct 2007	157/346	Sun Yat-sen University Cancer Center (SYSUCC)	319/448	The First Affiliated Hospital of Sun Yat-sen University (1st AH-SYSU)	NPC endemic
Data_GD2	Guangdong	Oct 2008–Jun 2013	168/222	SYSUCC	241/315	1st AH-SYSU	NPC endemic
Data_GD3	Guangdong	Oct 2008–Jun 2013	726/1065	Local hospitals in Guangdong province	880/1161	Local hospitals in Guangdong province	NPC endemic
Data_SD	Shandong	Oct 2008–Jun 2013	58/102	The Affiliated Hospital of Qingdao University, the Shandong Province Cancer Center	612/1543	Local hospitals in Shandong province	NPC non-endemic

In the same period, additional TB samples from NPC patients were collected from NPC endemic regions in Asia, including 122 samples from SYSUCC, 30 samples from the National Cancer Center of Singapore in Singapore, and 30 samples from the Chinese University of Hong Kong in Hong Kong. TB samples from patients with EBV-related malignancies were also collected, including 10 samples of gastric carcinoma from AH-QDU and 23 samples of lymphoma (Burkitt’s, NK/T cell, or Hodgkin’s) from SYSUCC. An additional 39 TW samples from patients with non-EBV-associated cancers were collected at SYSUCC. TW samples were also collected from healthy subjects in NPC non-endemic regions, including 83 samples from the Medical Examination Center of Henan Provincial Military Department in Henan Province, 100 samples from the Beijing Centers for Diseases Control and Prevention in Beijing, 116 samples from the Third People’s Hospital of Datong in Shanxi Province, and 11 Caucasian samples from the Karolinska Institute in Sweden and the VU University Medical Center in Netherlands.

The selection criteria for patients were self-reported Chinese and newly diagnosed patients without any radiotherapy, chemotherapy, or surgery. TW samples were collected before any treatment. Basic information was also collected from the participants regarding age, gender, residential region, ethnicity, and familial history of NPC or other cancers. Healthy controls with no self-reported history of cancer were randomly recruited from physical examination centers in hospitals and were frequency-matched to the cases by age (±5 years), gender, residential region, and ethnicity. This study was approved by the Human Ethics Committee at SYSUCC. Written informed consent was obtained from all the participants.

### Isolation of DNA

Genomic DNA from TW samples was prepared using a conventional method. Briefly, the subjects rinsed their mouths with 15 mL of 0.9% saline for 10 s. Buccal epithelial cells were pelleted by centrifugation at 5000×*g* for 10 min. The cells were re-suspended and digested in a lysis buffer [10 mmol/L Tris·HCl with pH 8.0, 100 mmol/L NaCl, 25 mmol/L ethylene diamine tetraacetic acid (EDTA), 0.5% Sarkosyl, and 0.1 mg/mL proteinase K] for 1–2 h at 55 °C. After treatment with RNase A, DNA was extracted from the cell lysate by adding phenol/chloroform and then precipitated with ethanol, followed by dissolving in 50 μL of water. Genomic DNA from TB samples and cells was extracted using a commercial DNA extraction kit (DNeasy Blood & Tissue Kit, Qiagen, Valencia, CA, USA).

### Sequence analysis and detection of SNP in *RPMS1*

**Fig. 1:**
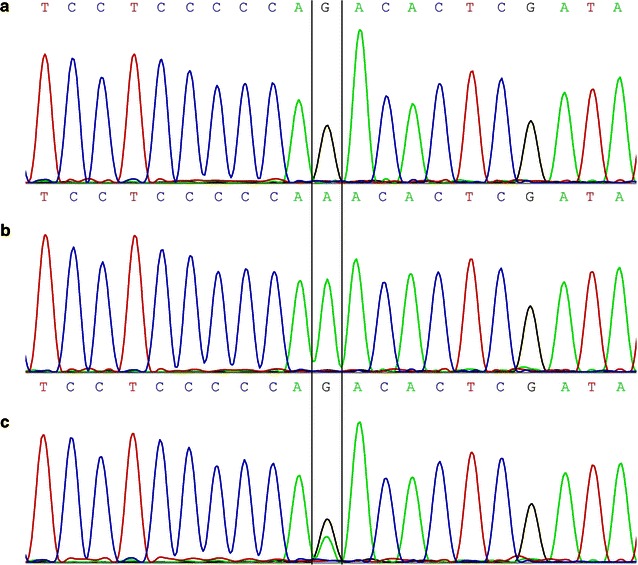
Validation of the *RPMS1* single nucleotide polymorphism (SNP) G155391A by Sanger sequencing. **a** Representative *RPMS1* 155391G variant (wild type). **b** Representative *RPMS1* 155391A variant (mutant type). **c** Representative mixture type, individuals infected with both *RPMS1* 155391G and 155391A variants

In the pilot study, sequences of *LMP1*, *EBNA1*, and the *BARTs* family were detected by standard PCR and the direct Sanger sequencing method [22]. For *RPMS1*, only the second coding exon was considered (sequence length approximately 282 bp, covering 89.74% of the *RPMS1* coding region), as there was no variation in the first exon according to pairwise comparisons among GD1, AG876, and two wild-type EBV genomes (GenBank Accession No. AY961628, DQ279927, AJ507799, and NC_007605). Considering the low number of DNA copies of EBV in the TW samples, three rounds of nested PCR were subsequently conducted to amplify the *RPMS1* fragment as a way to increase the detection rate. Three primer pairs are listed in Table 2. In the first round, 2 μL of each genomic DNA served as the template, and PCR was performed in a 25-μL reaction system containing 0.25 μL of 20 μmol/L primer pair RPMS1-1/2, 2.0 mmol/L magnesium chloride, 0.2 mmol/L of each dNTP, and 0.625 unit of *Go Taq* DNA polymerase (Promega, Madison, WI, USA). In the second round, 2 μL of mixture from the first round PCR was used as the template with the primer pair RPMS1-3/4 in a 25-μL reaction system. In the third round, the template was 5 μL of mixture from the second round PCR, using the primer pair RPMS1-5/6, in a 50-μL reaction system. Raji DNA and water were used as positive and negative controls, respectively. The amplification procedures for each round followed the manufacturer’s protocol. After PCR amplification, the nucleotide sequences of the PCR products were determined by Sanger sequencing (Fig. 1).

**Table 2:** Primers used in the nested polymerase chain reaction (PCR) and their sequences

Primer	EBV locus^a^	Sequences (5′ → 3′)	Note
EBNA1-1	96,750–67	GGGAAGTCGTGAAAGAGC	Outer primer
EBNA1-2	97,479–96	GGTGGAAACCAGGGAGGC	
EBNA1-3	97,052–72	GGTTTGGAAAGCATCGTGGTC	Inner primer
EBNA1-4	97,390–410	AACAAGGTCCTTAATCGCATC	
LMP1-CT-1	167,623–42	GCTAAGGCATTCCCAGTAAA	Outer primer
LMP1-CT-2	168,268–86	GATGAACACCACCACGATG	
LMP1-CT-3	167,755–72	CGGAACCAGAAGAACCCA	Inner primer
LMP1-CT-4	168,244–61	TCCCGCACCCTCAACAAG	
RPMS1-1	155,087–107	GCTGGGTTGATGCTGTAGATG	1st round nested
RPMS1-2	155,799–819	AGGGTCTGGACGTGGAGTTTG	
RPMS1-3	155,103–121	AGATGTGCCTGGCTCTGTC	2nd round nested
RPMS1-4	155,543–63	CAATGACTTTGTCACCTTTGG	
RPMS1-5	155,199–220	AGAAGGCGTAGAGCATGTCCAG	3rd round nested
RPMS1-6	155,460–81	GAGTACGACTGTGAGGTGGGCG	

*EBV* Epstein-Barr virus, *EBNA1* EBV nuclear antigen 1, *LMP1* latent membrane protein 1

^a^Coordinates relative to complete wild-type EBV genome (GenBank Accession No. NC_007605)

### Cell culture

NP69 is an immortalized human nasopharyngeal epithelial cell line originally presented by George Tsao at the University of Hong Kong and maintained at SYSUCC. NP69 cells were grown in defined Keratinocyte serum-free medium supplemented with epidermal growth factor (EGF) (Invitrogen, Grand Island, NY, USA). The purity of NP69 cells was verified using short tandem repeat (STR) markers with the Golden*e*ye™20A STR kit (Peoplespot Co., Beijing, China) and an ABI 3100 analyzer (Thermo Fisher Scientific, Grand Island, NY, USA). Raji and C666-1 cells were maintained at our laboratory and cultured in RPMI-1640 medium supplemented with 10% fetal bovine serum (Gibco, Grand Island, NY, USA). 293T cells were maintained at our laboratory and grown in Dulbecco’s modified eagle medium supplemented with 10% fetal bovine serum (Gibco). All cells were cultured in a humidified chamber with 5% CO_2_ at 37 °C.

### Plasmids and generation of stable *RPMS1* expression transfectants

Full-length cDNA of *RPMS1* was obtained by PCR from the cDNA library derived from the EBV-positive NPC cell line C666-1 and then cloned into the pBABE-Puro retroviral vector (Cell Biolabs, San Diego, CA, USA). Mutations were introduced using the Quick-Change Site-Directed Mutagenesis Kit (Stratagene, Santa Clara, CA, USA), and all mutations were verified by Sanger sequencing. The pBABE-Puro-RPMS1 (-Mut/-WT) expression vectors (constructed at our laboratory) and their corresponding control vectors (Cell Biolabs) were packaged into the retrovirus generated by 293 T cells, followed by the infection of NP69 cells. The respective stable transfectants in NP69 cells were selected against 1 μg/mL of puromycin.

### Western blotting

Western blotting was performed as described previously [30]. Briefly, cells were lysed in mammalian cell lysis buffer, and proteins within the clarified lysates were resolved by sodium dodecyl sulfate–polyacrylamide gel electrophoresis (SDS-PAGE) and transferred to polyvinylidene difluoride (PVDF) membranes for immunoblotting against the corresponding antibody. The results were revealed using enhanced chemiluminescent (ECL) detection reagents (Beyotime Co., Shanghai, China). The rabbit polyclonal anti-RPMS1 antibody was from Proteintech Group Inc. (Wuhan, Hubei, China), and the human anti-β-actin antibody was from Sigma-Aldrich Co. (St. Louis, MO, USA). A horseradish peroxidase (HRP)-conjugated anti-rabbit IgG antibody was used as the secondary antibody (Promega, Madison, WI, USA).

### Statistical analysis

To test the association between EBV variations and NPC risk, odds ratios (ORs) and 95% confidence intervals (CIs) were estimated by unconditional logistic regression. Subjects with the EBV prototype (155391G) were treated as the reference. ORs were adjusted for gender and age, where both were taken as categorical covariates (female or male; ≤35, 35–65, and >65 years). Fisher’s exact test was used to assess the frequency distribution of variables in two or more groups. The NPC risk associated with the affected EBV variations was characterized using the Cochran-Armitage trend test in the logistic regression analysis with adjustment for gender and age, where the variables of the EBV variations 155391G, 155391G/A, and 155391A were coded by 0, 1, and 2 in the statistical model, respectively. All statistical analyses were performed using the R3.0.1 software (http://www.r-project.org/). A *P* value of less than 0.05 was considered significant.

## Results

### Association between a SNP in the EBV genome and high risk of NPC

To identify genomic variations related to the NPC disease phenotype, in the pilot study, we sequenced the genomic regions of EBV-encoded genes, including *LMP1*, *EBNA1*, and the *BARTs* family, in 60 paired TW samples from NPC patients and healthy controls from a Cantonese population. Because, in NPC patients, multiple subtypes of EBV infection could be detected frequently in peripheral blood samples, and the EBV subtype detected in the normal nasopharyngeal tissues was more similar to the subtype in the TB samples [16, 22], we chose to sequence DNA extracted from the TW samples. We found one SNP in *RPMS1* (Loc155391 G>A) with a significant difference between the cases and controls, and all the subsequent experiments on larger sample sizes were then focused on this genomic variation. In contrast, no significant associations with NPC risk were observed at the *EBNA1* and *LMP1* loci (Table 3).

**Table 3:** Association between variations of *EBNA1* and *LMP1* in throat washing (TW) samples and the risk of NPC in Guangdong population (pilot study)

Gene	Subtype	NPC [no. (%)]	Healthy subjects [no. (%)]	*P*
*RPMS1*				<0.001
	155391G	8 (16.0)	29 (53.7)	
	155391G/A	0	4 (7.4)	
	155391A	42 (84.0)	21 (38.9)	
*EBNA1*				0.677
	V-val	45 (90.0)	44 (84.6)	
	P-ala	1 (2.0)	2 (3.8)	
	P-thr	2 (4.0)	1 (1.9)	
	Mix	2 (4.0)	5 (9.6)	
*LMP1*				0.080
	China 1	27 (64.3)	39 (68.4)	
	China 2	4 (9.5)	2 (3.5)	
	B95.8	2 (4.8)	10 (17.5)	
	Mix	9 (21.4)	6 (10.5)	

*Mix* mixture of two or more EBV subtypes. Other abbreviations as in Tables 1 and 2

In the discovery stage, TW samples from 157 NPC patients and 319 controls recruited from Guangdong Province were genotyped based on the 2nd exon sequence of *RPMS1* (Data_GD1; Table 1). The SNP was recognized as Loc155391 (G>A) based on its coordinates mapping to the wild-type EBV genome (GenBank Accession No. NC_007605). Logistic regression analysis with adjustment for age and gender revealed a strong association of the SNP at Loc155391 (named as G155391A) with a high risk of NPC (*P* < 0.001, OR = 4.47, 95% CI 2.71–7.37; Table 4).

**Table 4:** Single nucleotide polymorphism (SNP) G155391A of *RPMS1* and NPC risk

EBV variant	NPC	Healthy subjects	OR	95% CI	*P* value
Data_GD1					
155391G	28	132	1		
G155391A	10	40	1.53	0.66–3.54	0.321
155391A	119	147	4.47	2.71–7.37	<0.001
*P*_trend_^†^					<0.001
Data_GD2					
155391G	28	125	1		
G155391A	2	5	1.98	0.35–11.33	0.443
155391A	138	111	5.20	3.18–8.50	<0.001
*P*_trend_^†^					<0.001
Data_GD3					
155391G	99	357	1		
G155391A	12	84	0.61	0.32–1.17	0.136
155391A	615	439	5.27	4.06–6.85	<0.001
*P*_trend_^†^					<0.001
Data_SD					
155391G	40	560	1		
G155391A	0	18	0	0-Inf	0.987
155391A	18	34	7.52	3.69–15.32	<0.001
*P*_trend_^†^					<0.001
Overall^a^					
155391G	195	1174	1		
G155391A	24	147	0.92	0.56–1.51	0.746
155391A	890	731	5.27	4.31–6.44	<0.001
*P*_trend_^†^					<0.001
*I* ^2^			0		
*P* ^‡^					0.710

*OR* odds ratio, *95% CI* 95% confidence interval, *Inf* Infinity. Other abbreviations as in Tables 1 and 2

^a^Datasets integrated by meta-analysis

^†^ *P*_*trend*_ Cochran-Armitage trend test in logistic regression analysis with adjustment for age and gender

^‡^ *P* heterogeneity test among the four datasets

### Replication analyses

To replicate the association, Loc155391 was genotyped in two independent sample groups recruited from the same NPC endemic region, consisting of 168 NPC patients and 241 healthy controls from Data_GD2 and 726 NPC patients and 880 healthy controls from Data_GD3 (Table 1). Logistic regression analysis showed that SNP G155391A was significantly associated with a high NPC risk in both sample groups (Data_GD2: *P* < 0.001, OR = 5.20, 95% CI 3.18–8.50; Data_GD3: *P* < 0.001, OR = 5.27, 95% CI 4.06–6.85; Table 4), indicating that the strong association was replicated in the two independent sample groups. As further confirmation, logistic regression analysis for SNP G155391A was conducted in another sample group from Shandong Province in North China, which is a NPC non-endemic region, involving 58 NPC patients and 612 healthy controls (Data_SD). The result revealed a consistently strong association between SNP G155391A and a high NPC risk (*P* < 0.001, OR = 7.52, 95% CI 3.69–15.32; Table 4), indicating that the association was further replicated. Meta-analysis of all the four samples with a total of 1109 NPC patients and 2052 healthy controls showed that SNP G155391A was associated with a high risk of NPC among all tested regions (*P* < 0.001, OR = 5.27, 95% CI 4.31–6.44), and there was no evidence of heterogeneity among the included cohorts (*P* = 0.71; Table 4). In addition, no other variations of *RPMS1* were observed in any of the four sample groups.

### Association of *RPMS1* SNP G155391A and incidences of NPC and other malignancies

The frequencies of SNP G155391A were counted and compared among samples from Guangdong in South China, which is an NPC endemic region, as well as in North China and Europe, where NPC incidence is relatively low. High frequencies of SNP G155391A were detected among the controls from Guangdong (48.4%), whereas the frequencies were significantly lower in North China (1.2%–8.0%) and Europe (0) (*P* < 0.001; Table 5). The increasing trend in the frequency of SNP G155391A in samples from regions with low to high NPC incidence was consistently observed in NPC patients, using either TW or TB samples (both *P* < 0.001; Table 5). These results indicated that the frequency of SNP G155391A was associated with the NPC incidence and was significantly increased in the tumor tissues. Moreover, as Burkitt’s lymphoma, Hodgkin’s lymphoma, NK/T-cell lymphoma, and some gastric cancers are well known as EBV-related malignancies, we compared the distributions of the *RPMS1* SNP G155391A between other cancer samples and healthy controls. Interestingly, no evidence of association was observed between the *RPMS1* SNP G155391A and the risks of tested cancers except for NPC (*P* > 0.05; Table 6), suggesting that the association with the high-risk EBV variant might be specific to NPC.

**Table 5:** The frequencies of *RPMS1* SNP G155391A in NPC cases and healthy controls from various world regions

Sample	Source	Region	No. (sum)	G155391A			Frequency of G155391A (%)	NPC incidence	P*
				G	G/A	A			
NPC	TB	Shandong	36	23	0	13	36.1	Low	
		Guangdong	122	22	0	100	82.0	High	
		Hong Kong	30	2	0	28	93.3	High	
		Singapore	30	9	0	21	70.0	High	<0.001
	TW	Shandong	22	17	0	5	22.7	Low	
		Guangdong	1051	155	24	872	83.0	High	<0.001
Healthy subjects	TW	Europe	11	11	0	0	0.0	Low	
		Henan	83	81	1	1	1.2	Low	
		Beijing	100	91	1	8	8.0	Low	
		Shanxi	116	109	1	6	5.2	Low	
		Shandong	612	560	18	34	5.6	Low	
		Guangdong	1440	614	129	697	48.4	High	<0.001

*TB* tumor biopsy. Other abbreviations as in Tables 1 and 2

* Data combined in regions with low/high NPC incidence and probability calculated by Fisher’s exact test

**Table 6:** Association of *RPMS1* SNP G155391A with the risk of NPC and other malignancies

Region	Sample	Source	155391G & 155391G/A	155391A	OR	*P*
Guangdong	Healthy subjects^a^	TW	743	697	1	
	EBV-free tumor^#^	TW	24	15	0.67^†^	0.257
	NHL^‡^	TB	8	5	0.67^†^	0.582
	HL	TB	7	3	0.46^†^	0.345
	Lymphoma (NHL + HL)	TB	15	8	0.57^†^	0.213
	NPC	TB	22	100	8.35^˄^	<0.001
Shandong	Healthy subjects^a^	TW	578	34	1	
	EBVaGC	TB	10	0	0^†^	1.000
	NPC	TB	23	13	Inf^˅^	0.042

*NHL* non-Hodgkin’s lymphoma, *HL* Hodgkin’s lymphoma, *EBVaGC* EBV-associated gastric carcinoma. Other abbreviations as in Tables 1 and 2

^a^Healthy subjects from the discovery and replication stages were combined

^#^EBV-free tumors included lung cancer, liver cancer, colorectal cancer, and pancreatic cancer, among others, which were not associated with EBV

^‡^NHL included Burkitt’s and NK/T-cell lymphomas

^†^The frequency of *RPMS1* SNP G155391A in healthy subjects from the same region was considered as a reference, with Fisher’s exact test performed

^˄^Lymphoma was considered as a reference

^˅^EBVaGC was considered as a reference

### Functional characterization of *RPMS1* SNP G155391A

**Fig. 2:**
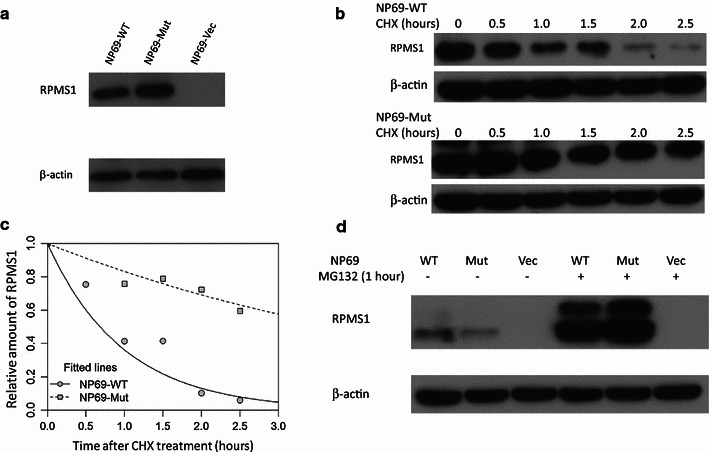
Effect of the *RPMS1* SNP G155391A on the degradation of RPMS1 protein. **a** Western blotting analysis showing the expression levels of RPMS1 in NP69 cell lines established with the stable integration of the pBABE-Puro retroviral vector of mutant *RPMS1* (-Mut), wild-type *RPMS1* (-WT), and control vector (-Vec), respectively. **b** Western blotting results showing the degradation of RPMS1 protein. NP69 cells with stable overexpression of mutant *RPMS1* (-Mut) or wild-type *RPMS1* (-WT) were incubated with 20 μg/mL cycloheximide (CHX) for the indicated periods of time (0, 0.5, 1.0, 1.5, 2.0, and 2.5 h). **c** Fitted curves of the degradation of the RPMS1 protein of EBV variations under the damped exponential model. **d** Western blotting results showing the RPMS1 protein expression in NP69 cells with stable overexpression of mutant *RPMS1* (-Mut) or wild-type *RPMS1* (-WT), treated with or without 10 μmol/L MG132 for 1 h

Endogenous RPMS1 protein was not detected, even though *RPMS1* was implicated in NPC development. Although the *BARTs* contain many EBV-encoded microRNA precursors [31], we failed to detect any alteration in the microRNAs predicted in the regions near *RPMS1* between the wild-type (155391G) and mutant (155391A) *RPMS1* (data not shown). Thus, we suspected that the variation of G155391A from guanine (G) to adenine (A), leading to the amino acid change from Asp (D) to Asn (N), might be related to *RPMS1* transcription or expression. Variations of the stable nasopharyngeal epithelial cell line NP69 integrating pBABE-Puro retroviral vector with mutant *RPMS1* (155391A), wild-type *RPMS1* (155391G), and empty vector, respectively, were successfully constructed as revealed by Western blotting (Fig. 2a). After cycloheximide (CHX) treatment, RPMS1 protein degradation was clearly proceeding after 0.5 h in the NP69 cells with wild-type *RPMS1* (155391G), whereas the degradation was hampered in the NP69 cells with mutant *RPMS1* (155391A) (Fig. 2b). Moreover, the damped exponential model indicated that the half-life for the mutant RPMS1 protein was significantly longer than that for the wild-type protein (3.2 vs. 0.6 h, *P* < 0.001; Fig. 2c), suggesting that the SNP G155391A is functionally regulating the protein stability of RPMS1. In addition, when treated with the proteasome inhibitor MG132, a significant increase in RPMS1 protein expression was observed in the stable NP69 cell lines with overexpression of either wild-type (155391G) or mutant *RPMS1* (155391A) (Fig. 2d), suggesting that the RPMS1 protein might be degraded through the ubiquitin–proteasome pathway.

## Discussion

In this multi-stage association study with a large sample size, we identified an EBV genomic sequence variation represented by *RPMS1* SNP G155391A that was associated with a high risk of NPC. This association is much stronger than those of non-viral environmental factors, such as the consumption of salted fish and preserved food, with NPC risk [32–34]. The frequency of *RPMS1* SNP G155391A was significantly associated with the NPC incidence, and higher frequencies were observed in the NPC endemic areas, suggesting that *RPMS1* SNP G155391A might explain the different incidences of NPC worldwide. *RPMS1* SNP G155391A was enriched in NPC patients but was not associated with other malignancies; these results support the hypothesis that there is a highly oncogenic EBV subtype specifically leading to NPC risk.

The identification of the high-risk *RPMS1* SNP G155391A for NPC emphasizes that the contribution of EBV strain variation to virus-associated malignancies should not be ignored. A similar scenario is the association of human papillomavirus (HPV) with cervical carcinomas, in which highly oncogenic HPV subtypes 16, 18, and 45 are the predominant contributors to the disease among more than 150 HPV subtypes [35, 36]. Therefore, HPV vaccine programs have shown promising population-level impacts, and the screening of HPV subtypes is important for the early detection of cervical carcinomas [37]. Indeed, serological EBV markers are potentially useful for screening individuals with a high risk of NPC in multiplex families [38]. The identification of the high-risk *RPMS1* SNP G155391A suggests that we should consider the contribution of EBV variations to the applications of serological EBV markers, such as DNA in NPC monitoring and prognostication [39]. With further investigation of other high-risk EBV variations, if any, we might be able to develop effective vaccines against high-risk EBV subtypes to promote NPC prevention.

*RPMS1* is a unique gene belonging to the EBV *BARTs* family, which is abnormally expressed in most NPC tissues at the RNA level and might contribute to NPC development [25, 26]. No endogenous RPMS1 protein has been reported in cultured NPC cells or NPC tumor biopsies [40], and thus, we suspected that *RPMS1* might be translated into protein at very low levels, or else that the RPMS1 protein was degraded very rapidly. Indeed, we found that the *RPMS1* variations defined by 155391A and 155391G are functionally relevant to the stability of RPMS1 protein overexpressed in vitro (Fig. 2). Compared with the low-risk 155391G, the high-risk 155391A resulted in a longer half-life of RPMS1 protein, as shown in the protein degradation assays. With oncogenic capacity, *RPMS1* has been shown to interact with the Notch intracellular domain and regulate the downstream pathway to promote cell differentiation and proliferation [41]. A recent genome sequencing study of NPC revealed accumulated mutations in the genes involved in the Notch pathway, including *NOTCH1*, *NOTCH2*, and *NOTCH3* [42], suggesting that the dysregulation of the Notch pathway might be an important driving event in NPC. These results further suggest that the interaction between EBV-encoded *RPMS1* and the host Notch pathway might be a significant process during NPC development and that the high-risk 155391A, leading to a longer half-life of RPMS1 protein, may exhibit stronger carcinogenesis potential.

## Conclusions

We discovered a high-risk EBV SNP for NPC, which suggests the existence of disease-related EBV subtypes. Moreover, our findings indicate that different distributions of EBV subtypes in different geographic regions and ethnic groups might be among the reasons for the differences in NPC incidence worldwide. Therefore, our results provide new insights for screening populations at a high risk of NPC and strategies for EBV vaccine development in the future. We acknowledge that further studies with larger sample sizes, more ethnic groups, and more geographic regions are needed to replicate our findings and rule out the confounding effects of population and the source of EBV, as the *RPMS1* SNP G155391A had much higher frequency in the Guangdong area based on TW samples. Certainly, more efforts are required to analyze the whole genome sequence of EBV to define haplotypes, instead of a single SNP, for genotyping the virus detected in healthy subjects or patients with different disorders and different ethnicities.
